# TRPM2 channels are not required for acute airway inflammation in OVA-induced severe allergic asthma in mice

**DOI:** 10.1186/1476-9255-10-19

**Published:** 2013-05-01

**Authors:** Adriana Sumoza-Toledo, Andrea Fleig, Reinhold Penner

**Affiliations:** 1 Laboratory for Cellular and Molecular Signaling, Center for Biomedical Research at The Queen’s Medical Center and John A. Burns School of Medicine, University of Hawai’i, 1301 Punchbowl St, Honolulu, HI, 96813, USA; 2 University of Hawaii Cancer Center, Honolulu, HI, 96813, USA

**Keywords:** Allergy, Asthma, Cytokines, Mouse model, TRPM2 channel

## Abstract

**Background:**

Airway inflammation and asthma have been linked to oxidative stress and the melastatin-related transient receptor potential cation channel, member 2 (TRPM2), which can be activated by reactive oxygen species (ROS), has emerged as a potential therapeutic target for inflammatory diseases.

**Objective:**

Using TRPM2 deficient (TRPM2^-/-^) mice, we investigated whether the TRPM2 ion channel, which mediates calcium (Ca^2+^) influx and lysosomal Ca^2+^ release, plays a role in the pathophysiology of severe allergic asthma in mouse.

**Methods:**

Severe allergic asthma was initiated in wild type (WT) and TRPM2^-/-^ mice by repeated sensitization with ovalbumin (OVA)/aluminum hydroxide on Days 0, 7 and 14, followed by intranasal challenge on Days 21, 22 and 23. Mice were investigated for the presence of airway responsiveness, airway inflammation, production of allergen-specific antibodies, cytokine response and lung pathology.

**Results:**

The absence of TRPM2 channels has no obvious effect on major etiologic markers of severe allergic asthma in this mouse model. Neither airway resistance nor mucus production are affected in TRPM2^-/-^ mice. TRPM2 channel ablation also does not alter airway inflammation or immunocyte infiltration and does not affect antibody response or cytokine levels.

**Conclusions:**

TRPM2 is not required for airway inflammation in OVA-induced severe allergic asthma in mice. Accordingly, TRPM2 might not be a suitable therapeutic target for airway inflammation caused by allergens in humans.

## Background

Asthma is a chronic airway inflammation characterized by intense eosinophil, mast cell, and lymphocyte infiltration, mucus hyper-production, and airway hyper-responsiveness [1]. Asthma symptoms develop when allergens activate antigen-specific helper T-lymphocytes (Th) to produce Th-2 cytokines, such as interleukin (IL)-4, IL-5, and IL13 [1]. Activated phagocytic cells (neutrophils, eosinophils, monocytes and macrophages) also play a role in the pathophysiology of airway inflammation due their release of large amounts of reactive oxygen species (ROS), lipid mediators, and cytokines [2,3]. Airway cells and tissues are also exposed to oxidative stress elicited by environmental pollutants (ozone, cigarette smoke, and dust), infections, inflammatory reactions or decreased levels of antioxidants that lead to enhanced levels of ROS [3,4]. It has been shown that ROS can damage DNA, lipids, proteins, and carbohydrates leading to impaired cellular functions and enhanced inflammatory reactions [3]. Consequently, it has been suggested that ROS play a role in airway disorders such as adult respiratory, distress syndrome (ARDS), cystic fibrosis, idiopathic fibrosis, chronic obstructive pulmonary diseases (COPD), and asthma [3,5].

The mammalian super family of transient receptor potential (TRP) cation channels can be subdivided into six subfamilies based on sequence homology: TRPC (canonical), TRPV (vanilloid), TRPM (melastatin), TRPA (ankyrin), TRPP (polycystin) and TRPML (mucolipin) [6,7]. TRP channels express in a broad range of cell types including sensory nerves, lung fibroblast, epithelial cells and immune cells [6,7]. Relevant to the context of asthma, TRPA1 channels have been implicated in pain and inflammatory responses in the airways in mice [8]. In addition, the TRPM2 channel has been implicated in stress-related inflammatory and neurodegenerative conditions [9-11]. However, the relevance of TRPM2 in severe asthma pathophysiology has not yet been explored.

TRPM2 is a non-selective calcium (Ca^2+^) influx and lysosomal Ca^2+^ release channel expressed in neutrophils [9,12], monocytes [9], Jurkat T cells [13], INS-1 cells [14] and mouse bone marrow derived-dendritic cells (BMDC) [15]. TRMP2 is co-activated by intracellular adenosine diphosphate ribose (ADPR) and Ca^2+^, downstream of ROS and chemokine signaling pathways [11,15-17]. TRPM2 activation by ADPR is further facilitated by the presence of nicotinic acid adenine dinucleotide phosphate (NAADP), cyclic ADPR (cADPR), and hydrogen peroxide (H_2_O_2_) [18-21], whereas adenosine monophosphate (AMP) and permeating protons (pH) negatively regulate TRPM2 activation [21-24].

TRPM2-deficient mice are more resistant to chronic experimental colitis due to defective chemokine (C-X-C motif) ligand 2 (CXCL2) production by monocytes and reduced neutrophil infiltration [9]. Yet, a recent publication has suggested no role for TRPM2 channel in chronic obstructive pulmonary disease [25]. Intriguingly, cADPR induces Ca^2+^ release in airway smooth muscle (ASM) [26,27] and acetylcholine (ACh) and endothelin-1 (ET-1) are considered to regulate airway caliber through cADPR-mediated Ca^2+^ release in these cells [28]. Moreover, mice that lack CD38, an ectoenzyme that generates free ADPR through the hydrolysis of nicotinamide adenine dinucleotide (NAD^+^) and its cADPR glycohydrolase activity, exhibit altered airway responsiveness to methacholine [28,29].

In the present study, we assessed the role of TRPM2 channels in airway inflammation by using an experimental OVA-induced severe asthma model. We found that airway responsiveness, airway inflammation, production of allergen-specific antibodies, and cytokine response were unaffected in TRPM2^-/-^ mice when compared to OVA-sensitized and challenged WT mice. Our findings suggest that in this experimental model the TRPM2 channel is not required for airway inflammation to occur.

## Methods

### Animals

C57BL/6*trpm2*^*+/+*^(wild type; WT) and C57BL/6 *trpm2*^*-/-*^(knock out; TRPM2^*-/-*^) mice were bred and housed under pathogen free conditions. TRPM2^*-/-*^mice were generated as previously described [9]. All mice were genotyped by PCR before the experiments to confirm disruption of *trpm2*^*-/-*^ gene. Mice were 8-12 weeks old at the time of the experiments. All protocols involving rodents were reviewed and approved by The Institutional Laboratory Animal Care and Use Committee (IACUC) at The University of Hawaii and The University of California, San Francisco.

### Allergen sensitization and challenge of mice

Sensitization and challenge of mice were performed as previously described [30]. Briefly, TRPM2^-/-^ mice and WT littermate were sensitized intraperitoneally with 50 μg ovalbumin (OVA; grade V; Sigma-Aldrich) plus 1 mg Alum (Sigma-Aldrich) in 200 μl 0.9% sodium chloride (saline; Hospira) on Days 0, 7, and 14. On Days 21, 22 and 23, mice were anesthetized with isoflurane (Hospira) and challenged with 100 μg OVA in 50 μl saline by nasal administration. Control groups were treated identically except OVA was missing in the solutions. Mice were euthanized and studied on Day 24.

### Measurement of airway hyper-responsiveness

Airway resistance in response to intravenously administered acetylcholine was measured using a flexiVent system (SCIREQ, Montreal, Canada) as previously described [30]. Mice were anesthetized with ketamine (100 mg/kg) and xylazine (10 mg/kg) and acepromazine (2-3 mg/kg); paralyzed with pancuronium (0.1 mg/kg intraperitoneally), intubated with a 20G cannula and mechanically ventilated at a frequency of 150 breaths per minute and 2 cmH_2_O positive end-expiratory. Lung resistance was measured at baseline and in response to increasing intravenous doses of acetylcholine (0, 0.1, 0.3, 1, 3 and 9.6 μg/g body weight) using the linear single compartment model.

### Bronchoalveolar lavage fluid (BAL) leukocytes count

Lungs from sacrificed mice were flushed once with 1 ml PBS/1% fetal calf serum (FCS) to obtain bronchoalveolar lavage (BAL) fluid. The total number of cells was determined by a hemocytometer. A maximum of 2 × 10^5^ cells were centrifuged on a microscope slide and stained with Diff-Quick (Polyscience). Differential cell counts were made at 3400 magnification, and at least 100 cells were counted per slide.

### Histology and immunohistochemistry

For histologic analysis of goblet cell hyperplasia, tissue samples were fixed in 4% phosphate-buffered formalin, embedded in paraffin, cut into 5-7 μm sections and stained with periodic acid-Schiff (PAS) reagent (Sigma-Aldrich) following manufacturer instructions. To evaluate inflammatory infiltration, tissue sections were stained with hematoxylin and eosin. Scoring was performed at 200x magnification by examining 40 consecutive fields of the peribronchiolar, perivascular, and alveolar areas. Mast cells were counted at 20x magnification in lung sections stained with toluidine blue.

### Detection of serum IgE antibodies

Blood samples were collected using the heart puncture method and serum was separated by centrifugation for 15 minutes at 6000 g. OVA-specific IgE antibodies were measured in a serum dilution series by endpoint titration enzyme-linked immunosorbent assay (ELISA). Briefly, plates were coated with 1 mg/ml OVA and alkaline phosphatase-conjugated anti-mouse isotype specific antibodies (Southern Biotechnology) and 4-nitrophenyl phosphate (Sigma-Aldrich) were used for detection. Absorbance was measured at 405 nm with 492 nm as a reference wavelength.

### Cytokine levels

The concentration of interleukin IL-5, IL-6, IL-10, IL-13 and transforming growth factor beta 1 (TGFB1) in the BAL fluid of five independent OVA and saline treated WT and TRPM2^-/-^ mice were measured using the specific Single Analyte ELISArray™ Kit (Qiagen) following manufacturer instructions. Samples were analyzed at 450 nm using a Benchmark plus microplate reader spectrophotometer (BioRad).

### Statistical analysis

Data are reported as mean ± SEM. Significance testing was performed by Student’s paired *t*-test for significant differences between two groups and ANOVA to test significant differences among the groups of mice. A value of p < 0.05 was considered to be statistically significant.

## Results

### Airway resistance and mucus production are not affected in TRPM2^-/-^ mice

**Figure 1:**
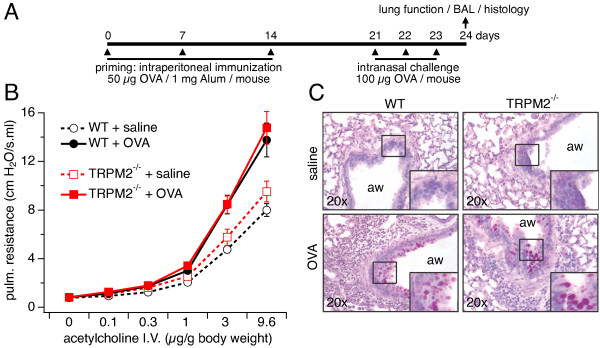
**Airway resistance and mucus production are not affected in TRPM2**^**-/- **^**mice. A**) General priming and challenge scheme for the experimental allergic model used in all investigations (see Methods for details). **B**) Airway resistance to increasing concentrations of intravenously administered acetylcholine in WT (black symbols) and TRPM2^-/-^ (red symbols) mice challenged with OVA and saline. Values represent means ± SEM (n = 10) from 2 experiments with 5 mice per group. ACh concentrations of 3 μM or higher produced significantly higher resistance in OVA-treated mice compared to saline-treated, but no significance differences were observed between WT and TRPM2^-/-^ mice. **C**) PAS staining of lungs from WT (left panels) or TRPM2^-/-^ mice (right panels) challenged with saline (top panels) or OVA (bottom panels). Images are representative of 10 mice per group. Relevant areas of normal and inflamed tissue with mucus-producing goblet cells are marked by a square and shown as enlarged insets in each graph (aw = airway).

Given that airway inflammation and asthma have been linked to oxidative stress [2], we investigated whether TRPM2, a Ca^2+^-permeable ion channel involved in ROS signaling, contributes to pathophysiology. For this, we analyzed the inflammatory allergic reaction of WT and TRPM2^-/-^ mice in an OVA-induced severe asthma model. Airway reactivity was measured in WT and TRPM2^-/-^ mice following OVA or saline challenge. As illustrated in Figure 1B, we observed an increase in airway resistance in both OVA-challenged WT and TRPM2^-/-^ mice when assessing responses to increasing doses of acetylcholine (filled symbols) compared to saline-challenged WT and TRPM2^-/-^ mice (open symbols), indicating the development of airway hyper-reactivity (AHR). Thus, the airway resistance in OVA-treated WT and TRPM2^-/-^ mice was significantly increased with no significant differences between the mouse strains.

Hyperplasia of mucus-producing goblet cells in the lung is also a characteristic of allergic asthma [1,31]. Therefore, we investigated the hyperplasia of goblet cells by histologic examination of PAS-stained lungs from OVA- and saline-sensitized and challenged WT and TRPM2^-/-^ mice. We observed that OVA-sensitized WT and TRPM2^-/-^ mice showed similar severity of goblet cell hyperplasia (Figure 1B, bottom panels; see magnified inset), while the saline group showed no signs of increased hyperplasia (Figure 1B, top panel). Hence, absence of TRPM2 seems to have no significant influence on airway resistance and mucus production.

### TRPM2 channel ablation does not alter allergic airway inflammation

**Figure 2:**
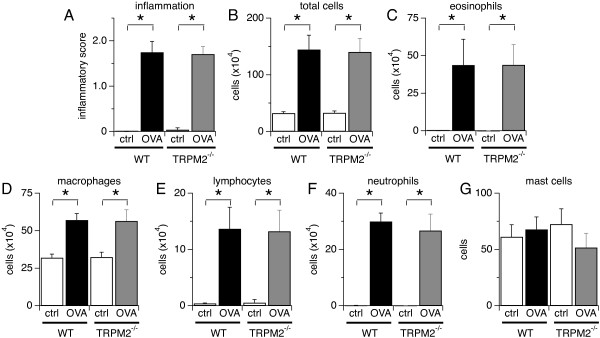
**TRPM2 channel ablation does not alter allergic airway inflammation. A**) Ranked inflammation scores for alveolar, peribronchiolar and perivascular regions of the lung, prepared by staining with hematoxylin and eosin, from OVA and saline-challenged WT and TRPM2^-/-^ mice. **B**-**F**) BAL cells were collected, identified and enumerated microscopically using Diff-Quick stained preparations. Total cell numbers (**B**) and numbers of eosinophils (**C**), macrophages (**D**), lymphocytes (**E**), and neutrophils (**F**) in the BAL fluid, and mast cells (**G**) in toluidine blue-stained lung tissue of WT and TRPM2^-/-^ mice challenged with saline or OVA. Except for mast cells, cell numbers were significantly increased by OVA challenge in both WT and TRPM2^-/-^ mice, but no significant difference was seen between WT and TRPM2^-/-^ mice. Data are shown as means ± SEM from 10 mice per group (* indicates p < 0.05).

It has been shown that TRPM2 channels control neutrophil infiltration in a mouse model of colitis by regulating CXCL2 chemokine production in monocytes [9]. BMDC from TRPM2^-/-^ mice also exhibit compromised chemotaxis towards CXCL12 and CCL19 chemokines [15]. To further evaluate whether TRPM2 contributes to airway inflammation and severe asthma, we examined whether the recruitment of inflammatory cells into the lungs was affected in the absence of TRPM2 channels. To assess the inflammatory response, we scored the histological changes in the lung parenchyma of OVA- and saline-challenged WT and TRPM2^-/-^ mice. As illustrated in Figure 2A, OVA induced significant inflammatory cell infiltration into the lung parenchyma in both WT and TRPM2^-/-^mice (filled bars) compared to mice that received saline only (open bars); however, no difference was observed between the two mouse strains. In addition, the total number of cells in the BAL fluid of OVA-challenged WT mice was significantly higher than in the control group challenged with saline, however, this did not differ from the total number of cells recovered in BAL fluid from OVA-challenged TRPM2^-/-^ mice (Figure 2B). The differential cell count revealed that eosinophils were the main cell type in BAL fluid (Figure 2C), as is characteristically observed in allergic airway inflammation, yet no significant difference was found between the mice strains. Cell counts of macrophages (Figure 2D), lymphocytes (Figure 2E), and neutrophils (Figure 2F) were not different in the BAL fluid from OVA-challenged WT and TRPM2^-/-^ mice (Figure 2D). In contrast, mast cell counts in the lung tissue of OVA-challenged mice were not increased in either group and WT and TRPM2^-/-^ remained similar (Figure 2E). Together, these data indicate that the TRPM2 channel does not regulate the bronchoalveolar inflammatory cell infiltration in an OVA-induced severe allergic asthma mouse model.

### Absence of TRPM2 does not alter antibody response and cytokine levels

**Figure 3:**
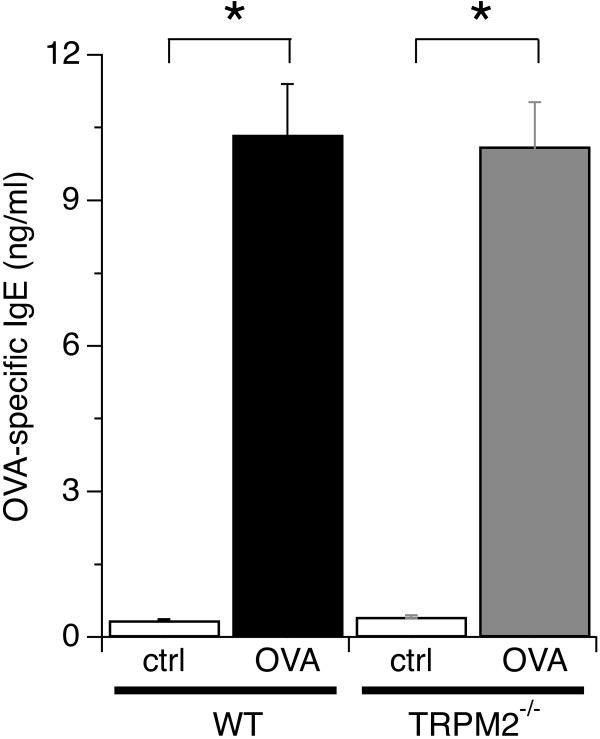
**Allergen-specific antibody responses of WT and TRPM2**^**-/- **^**mice are similar.** Serum OVA-specific IgE antibodies in WT and TRPM2^-/-^ mice following saline or OVA treatment and measured by endpoint ELISA. OVA-IgE antibodies were significantly increased in both OVA-treated WT and TRPM2^-/-^ mice, but no significant difference was seen between WT and TRPM2^-/-^ mice. Data represent mean values ± SEM from 10 mice per group (* indicates p < 0.05).

**Figure 4:**
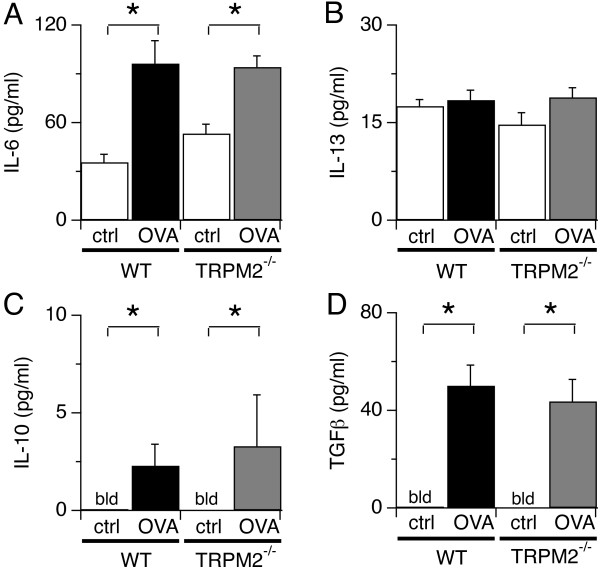
**Cytokine response is not modified in the absence of TRPM2 channels.** Levels of IL-6 (**A**), IL-13 (**B**), IL-10 (**C**), and TGFβ1 (**D**) in the BAL fluid of OVA- and saline-treated WT and TRPM2^-/-^ mice (bld = below levels of detection). Significant elevations in the level of cytokines were detected following OVA treatment in WT and TRPM2^-/-^ mice compared to saline control group (ctrl), but no significant difference was found between mouse strains. Values represent pooled data ± SEM from 5 mice per group (* indicates p < 0.05).

We next investigated whether antibody responses to allergens were affected in TRPM2^-/-^ mice. For this, we determined serum levels of OVA-specific IgE antibodies in WT and TRPM2^-/-^ mice treated with OVA or saline. Figure 3 shows a significant increase in OVA-specific IgE antibodies in OVA-challenged WT and TRPM2^-/-^ mice compared to saline treated mice with no apparent difference between the two mice strains after repeated exposure to OVA. Since TRPM2 can also regulate cytokine secretion [9], we examined whether Th2-type cytokines were affected by disrupted TRPM2 channels. We measured levels of IL-5, IL-6, IL-10, IL-13 and TGFB1 in the BAL fluid of OVA- and saline-treated WT and TRPM2^-/-^ mice. The results revealed that OVA challenge evoked significant and comparable levels of IL-6, IL-10, TGFB1 and IL-13 cytokines in WT and TRPM2^-/-^ mice (Figure 4). IL-5 levels were not detectable in mice treated with saline or OVA (data not shown), possibly due to detection limits of the experimental kit used. In summary, these data indicate that TRPM2 channels are not involved in the pathophysiology of OVA-induced severe allergic asthma in our mouse model.

## Discussion

Allergen-induced airway inflammation is an eosinophilic inflammation and type 2 T and B lymphocytes-driven response [1]. In this study, we investigated the role of TRPM2 channels in the pathophysiology of severe asthma by using TRPM2-deficient mice in a model of OVA-induced inflammatory airway disease. Our data indicate that TRPM2 channels are not required for acute airway inflammation to occur. Deletion of TRPM2 did not affect airway resistance (Figure 1B), mucus production (Figure 1C), inflammatory cell infiltration (Figure 2), allergen-induced production of IgE (Figure 3), and cytokine production (Figure 4) following allergen treatment. This is somewhat surprising in light of previous studies that used the dextran sulphate sodium (DSS)-induced chronic experimental colitis mouse model [9] and the lipopolysaccharide (LPS)-induced lung inflammation mouse model [32]. In the colitis model, the TRPM2 channel controls CXCL2 production in monocytes and consequently affects neutrophil infiltration, and in the lung inflammation model, TRPM2 plays a protective role by preventing ROS production in neutrophils. DSS induces inflammatory bowel disease-like colitis in mice by causing toxicity in colonic epithelial cells of the basal crypts [33]. Moreover, the inflammatory response is mediated by monocyte-dependent chemokine production in response to locally generated ROS [9]. Interestingly, LPS may regulate lung inflammation via Toll-like receptor 4 (TLR4) signaling in alveolar macrophages [34], whereas allergen-induced airway inflammation is driven by type 2 T lymphocytes and cytokines [1]. In contrast to the above studies, a recent study of chronic obstructive pulmonary disease (COPD) showed no role for TRPM2 in airway inflammation in mice exposed to ozone, LPS or tobacco smoke [25]. The CD8+ cell is the accepted crucial lymphocyte subtype in COPD. Therefore, TRPM2 appears to be preferentially involved in chronic inflammatory responses with a strong phagocytic cell component and might not have a prominent role in acute inflammatory processes. It also suggests that redundant mechanisms, including other ion channels, might compensate for the absence of TRPM2 channels during certain inflammatory processes. Other chronic inflammatory models should be used to address this hypothesis, including models of chronic allergen exposure or using alternative allergens such as dust mite and cockroach extracts.

The TRPM2 channel is expressed in the plasma membrane of neutrophils and monocytes/macrophages [9] and in lysosomes of BMDC [15] and controls their chemotaxis or cytokine production by regulating intracellular calcium concentration upon cell activation. TRPM2-deficient neutrophils exhibit defective *in vitro* chemotactic responses and calcium signals toward N-formyl-methionine-leucine-phenylalanine (fMLP), a peptide chain produced by some bacteria [9], although CXCL2-mediated responses remained unaffected. TRPM2^-/-^ neutrophils are also defective in ROS production [32]. In our model of allergen induced-chronic inflammation, deletion of TRPM2 expression did not affect IL-6, IL-10, IL-13 and TGFβ1 production (Figure 4) or inflammatory cell infiltration into the airway (Figure 2).

A central mediator of asthma is the IgE antibody, which is produced by sensitized allergen-specific B cells [1]. Allergens increase IgE levels in the serum of susceptible subjects subsequent to stimulation [1]. IgE antibodies then bind to the high-affinity IgE receptor, Fc epsilon receptor I (FcϵRI), present in mast cells, eosinophils, and basophils, thereby sensitizing these cells to allergen exposure [1]. IgE-FcϵRI complexes trigger degranulation of cytoplasmic vesicles containing histamine and *de novo* formation of eicosanoids and ROS in mast cells, eosinophils, and basophils, resulting in smooth muscle contraction [1]. Our data indicate that TRPM2 channels have no direct effect on allergen-induced production of IgE (Figure 3).

Airway caliber regulation by ACh and ET-1 occurs through a yet to be elucidated mechanism involving cADPR-mediated Ca^2+^ release in ASM cells [28]. It is known that cADPR activates ryanodine receptors [35] and can facilitate TRPM2 activation [36]. In addition, airway responsiveness to methacholine is altered in the absence of CD38, an ectoenzyme that generates free ADPR and cADPR from NAD^+^[28,29]. In our study, we observed no difference in airway resistance between TRPM2-deficient mice and WT mice challenged with OVA, suggesting that ACh does not require TRPM2 activity to regulate airway caliber. Interestingly, another TRP channel, TRPA-1, appears relevant to allergen-induced asthma models, since TRPA-1 knockout mice were shown to be more resistant to airway inflammation and hyperactivity than WT mice [8].

## Conclusions

In conclusion, although TRPM2 appears to be involved in certain inflammatory responses that are mediated by monocyte-dependent chemokine production in response to ROS, this inflammatory mechanism may not dominate the mechanisms engaged in the OVA-mediated severe airway inflammation model. Hence, this model might not be suitable to test for TRPM2 as a therapeutic target for allergen-induced airway inflammation or the channel itself might not play a significant role in allergen-mediated inflammation. Nevertheless, further investigations using a less severe asthma model are needed to fully address the role of TRPM2 in lung inflammation.

## Abbreviations

TRP: Transient receptor potential cation channels; TRPM2: Melastatin-related transient receptor potential cation channel member 2; TRPM2-/-: TRPM2 deficient mice; WT: TRPM2 wild type mice; Ca2+: Calcium; ADPR: Adenosine diphosphate ribose; cADPR: Cyclic ADPR; NAADP: Nicotinic acid adenine dinucleotide phosphate; H2O2: Hydrogen peroxide; AMP: Adenosine monophosphate; NAD+: Nicotinamide adenine dinucleotide; ROS: Reactive oxygen species; OVA: Ovalbumin; BAL: Bronchoalveolar lavage fluid; ASM: Airway smooth muscle; FcϵRI: Fc epsilon receptor I; ACh: Acetylcholine; ET-1: Endothelin-1; LPS: Lipopolysaccharide; fMLP: N-formyl-methionine-leucine-phenylalanine; CXCL2: Chemokine (C-X-C motif) ligand 2; BMDC: Bone marrow derived-dendritic cells; PAS: Periodic acid-Schiff; DSS: Dextran sulphate sodium; Th: Helper T-lymphocytes; TLR4: Toll-like receptor 4; IL: Interleukin.

## Competing interests

The authors declare that they have no competing interests.

## Authors’ contributions

All authors conceived and designed the study. A.S-T, acquired, analyzed and interpreted data and wrote manuscript. R.P., A.F., interpreted data and wrote the paper. All authors read and approved the final manuscript.
